# Prevalence and cumulative incidence of abnormal cervical cytology among HIV-infected Thai women: a 5.5-year retrospective cohort study

**DOI:** 10.1186/1471-2334-11-8

**Published:** 2011-01-07

**Authors:** Amphan Chalermchockcharoenkit, Chenchit Chayachinda, Manopchai Thamkhantho, Chulaluk Komoltri

**Affiliations:** 1 Department of Obstetrics and Gynaecology, Faculty of Medicine Siriraj Hospital, Mahidol University, Thailand; 2 Division of Clinical Epidemiology, Faculty of Medicine Siriraj Hospital, Mahidol University, Thailand

## Abstract

**Background:**

Cervical cancer is one of the most common AIDS-related malignancies in Thailand. To prevent cervical cancer, The US Public Health Service and The Infectious Disease Society of America have recommended that all HIV-infected women should obtain 2 Pap smears 6 months apart after the initial HIV diagnosis and, if results of both are normal, should undergo annual cytological screening. However, there has been no evidence in supporting whether this guideline is appropriate in all settings - especially in areas where HIV-infected women are living in resource-constrained condition.

**Methods:**

To determine the appropriate interval of Pap smear screenings for HIV-infected Thai women and risk factors for subsequent abnormal cervical cytology, we assessed the prevalence, cumulative incidence and associated factors of cervical cell abnormalities (atypical squamous cell of undetermined significance or higher grades, ASCUS+) among this group of patients.

**Results:**

The prevalence of ASCUS+ was 15.4% at the first visit, and the cumulative incidence of ASCUS+ gradually increased to 37% in the first 3.5 years of follow-up appointments (first 7 times), and tended to plateau in the last 2 years. For multivariate correlation analysis, women with a CD4 count <350 cells/μL had a significant correlation with ASCUS+ (*P *= 0.043). There were no associations of subsequent ASCUS+ with age, pregnancy, contraceptive method, highly active anti-retroviral treatment, assumed duration of infection, or the CD4 count nadir level.

**Conclusion:**

There are high prevalence and cumulative incidence of ASCUS+ in HIV-infected Thai women. With a high lost-to-follow-up rate, an appropriate interval of Pap smear screening cannot be concluded from the present study. Nevertheless, the HIV-infected Thai women may require more than two normal semi-annual Pap smears before shifting to routinely annual cytologic screening.

## Background

Thailand is one of the world's endemic areas for HIV infection. In 2007, approximately 250,000 women were living with HIV infection[1]. Women living with HIV-AIDS clearly have an increased risk of cervical cancer. A recent study in HIV-infected women in Thailand found that cervical cancer was the most common AIDS-related malignancy[2]. Sufficient Papanicolaou (Pap) smear screening would render a high yield in early detection because of its affordability, availability and accessibility. Cervical squamous cell abnormalities in HIV-infected women, compared with women who are not infected, progress more rapidly to more significant cervical intraepithelial neoplasia (CIN) or even invasive cervical cancer[3].

Our previous study indicated that 13.3% of HIV-infected pregnant women had cervical squamous cell abnormalities[4], while the prevalence of abnormal Pap smears from many studies seemed to be higher (20%-40%)[5-8]. However, there are different backgrounds. Many studies reported that cytology was primarily subject to false negative results[3,7,9-12]. Even with a negative for intraepithelial lesion (NIL) with an initial Pap smear, 20% of HIV-infected women will be later found with biopsy-confirmed cervical squamous cell intraepithelial lesions (SILs) in 3 years[3]. This may either reflect an incident lesion or reflect a prevalent lesion missed on Pap smear. Moreover, a study aimed at evaluating the sensitivity and specificity of Pap smears showed that 38% of all CIN would have been missed if routine colposcopy and biopsy had not been performed[7].

HIV treatment guidelines issued by The US Public Health Service and The Infectious Disease Society of America have recommended that all HIV-infected women should obtain two Pap smears 6 months apart after an initial HIV diagnosis and, if the results of both are normal, these women should then undergo annual cytologic screening[13]. However, the guideline has not been revised since 1995 and there has been no evidence to support that this guideline is appropriate in all settings, especially for HIV-infected women living in resource-constrained conditions where long-term studies are difficult to conduct. Moreover, currently no national cervical screening guidelines are in use in Thailand.

For these reasons, semi-annual Pap smear screenings for all HIV-infected Thai women, (not only women with CD+ 4 counts <200 cells/μL), development of health education and improved efforts to increase the trust between health care providers and women, have been implemented in our clinic since 2004. The present study aimed to determine an appropriate strategy for Pap smear screening and risk factors related to subsequent abnormal cervical cytology. Prevalence, cumulative incidence and associated factors of cervical cell abnormalities (atypical squamous cell of undetermined significance; ASCUS or higher grades, i.e. atypical squamous cells cannot exclude high grade squamous intraepithelial lesion; ASC-H, low grade squamous intraepithelial lesion; LSIL, High grade squamous intraepithelial lesion; HSIL, squamous cell carcinoma) designated as ASCUS+ among this group of patients were assessed.

## Methods

With the program implementation of semi-annual Pap smear screening for all HIV-infected women at the Female Sexually Transmitted Disease Clinic (STD Clinic), Faculty of Medicine Siriraj Hospital, Mahidol University from January 2004 to December 2009, the STD-medical records of 901 HIV-infected women were available for review to search for the results from first Pap smears. In our clinical protocol, all HIV-infected women were counselled to receive baseline Pap smear screening, and were required to come back for the next screening every 6 months. Sociodemographic data were collected using a structured medical record form. Blood sample for CD4+ count was obtained every 6 months. The result was determined in a local laboratory conducted by the Department of Microbiology in our hospital.

After a clinical and pelvic examination, women with signs of STDs were counselled and treated, and asked to return to the clinic 2 weeks later for baseline Pap smear screening. All Pap smear specimens were obtained by gynaecologists from the endocervix, cervical transformation zone and discharge at posterior fornix of vagina using a cotton tip stick and Ayre spatula, as described in the VCE technique. Cytological analyses were undertaken at the Division of Cytology of Department of Obstetrics and Gynaecology, Faculty of Medicine Siriraj Hospital. All Pap smear tests were processed and read by our certified senior cytotechnologists based on the 2001 Bethesda system guideline[14]. According to our policy, colposcopy must be offered in all cases of ASCUS+, but due to the occurrence of lost to follow-up, some of them were not able to have colposcopy. If indicated by colposcopy or cytology results, lesions were further evaluated by biopsy, endocervical curettage, or loop electrical excision. Definite surgical treatment (hysterectomy) was provided as indicated[15].

Participants who initially had NIL and came for their second Pap smear screening were included in the subgroup analysis to assess the cumulative incidence and factors associated with ASCUS+: namely age, parity, abortion, contraceptive methods, antiretroviral treatment, assumed duration HIV infection, CD4 count nadir and baseline CD4 count. All women with subsequent ASCUS/LSIL were not repeatedly enrolled among the rest of the study population. The assumed duration HIV infection was the duration that was estimated by the patient after counseling and the CD4 count nadir was the lowest CD4 count recorded. Data entry and analysis was performed by SPSS version 13 (SPSS, Chicago, IL, USA). Data were presented as frequency, percentage, mean ± standard deviation (SD), or median with ranges as appropriate. The cumulative incidence of ASCUS+ was estimated over the course of this study using standard life table methods. The student's *t*-test was used to compare means and Pearson *X*^2^-test or Fisher's exact test was used to compare proportions between HIV-infected women with ASCUS+ and NIL at first Pap smear, and between HIV-infected women with subsequent ASCUS+ and NIL who had a NIL at first Pap smear. Statistically significant differences were defined as *p *< 0.05. The Mann Whitney *U *test was used for variables that were not normally distributed. Multivariate correlation analysis was used to adjust for potential confounding factors. The study was conducted in accordant with the ethics principle of declaration of Helsinki, and the study protocol was approved by the Siriraj Institutional Review Board.

## Results

Of 901 HIV-infected women, 80 women were excluded as 18 women did not received Pap smear screening, 12 women did not have an intact cervix at baseline, 31 women had a previous diagnosis or treatment of cervical intraepithelial neoplasia, and 19 women had no documentation of CD4+ count. As a result, 821 women were included in the analysis. The study population had a mean age of 30.1 years (range 14 - 65), a median baseline CD4 count of 324 cells/μL (range 2 - 999) and a median CD4 count nadir of 206 cells/μL (range 2 - 930).

Of the 821 women, 237 women (28.9%) had CD4 counts less than 200 cells/μL and 395 women (48.1%) were receiving highly active antiretroviral therapy (HAART). There were 443 women who initially came to the clinic for antenatal care (ANC), 43 women for postpartum Pap smear screening, 37 women with STD related problems or positive pre-operative gynaecologic anti-HIV screening, and 298 women referred from the HIV clinic, Department of Preventive Medicine, for Pap smear screenings. The Pap smear screening data were from the women's first 12 semi-annual visits (over a period of 5.5 years) and consisted of 2,852 Pap smear tests.

### Prevalence of ASCUS+

The prevalence of cervical squamous cell abnormalities from 821 initial Pap smear screenings was 15.4%, consisting of ASCUS 2.8%, atypical squamous cells cannot exclude high grade squamous intraepithelial lesion (ASC-H) 0.6%, LSIL 8.5%, and HSIL 3.5% (Table 1). The most common coincident genital infection was fungal infection at 19.4% of the women. Compared with women with NIL, those with ASCUS+ had significantly higher proportions of receiving HAART and the baseline CD4 count <200 cells/μL (58.3% vs. 46.3%, 37.8% vs. 28.2%, respectively). A higher proportion of baseline CD4 count <350 cells/μL was also found in women with ASCUS+ (66.9% vs. 52.4%). In addition they had a significantly longer mean of assumed duration of HIV infection but a lower mean of CD4 count nadir (7.1 ± 4.1 years vs. 5.9 ± 3.4 years and 188.9 ± 142.7 cells/μL vs. 239.9 ± 182.8 cells/μL, respectively) as shown in Table 2.

**Table 1:** Frequency of cervical cytological findings at the first visit

Cervical cytological findings	Frequency (cases)	Percent
**Negative for intraepithelial lesion**		
Without organism and inflammation	412	50.2
Candida and budding yeasts	159	19.4
*Trichomonas vaginalis*	13	1.6
Bacterial vaginosis	2	0.2
Herpes simplex virus	12	1.5
Reactive cellular changes associated with inflammation	96	11.7
**Squamous cell abnormalities**		
ASCUS	23	2.8
ASC-H	5	0.6
LSIL	70	8.5
HSIL	29	3.5
**Total**	821	100

ASCUS, atypical squamous cell of undetermined significance; ASC-H, atypical squamous cell cannot exclude HSIL; LSIL, low grade squamous intraepithelial lesion;

HSIL, high grade squamous intraepithelial lesion.

**Table 2:** Comparison of the baseline characteristics of women with ASCUS+ and NIL at first Pap smear

	ASCUS+ (N = 127)	NIL (N = 694)	*P*
**Age (yrs)**	30.9 ± 8.4	29.8 ± 7.1	0.080
**Gravidarum ≥ 1**	102 (80.3)	592 (85.3)	0.153
**Parity ≥ 1**	93 (73.2)	526 (75.8)	0.537
**Abortion ≥ 1**	37 (29.1)	226 (32.6)	0.446
**Contraception**			
Tubal sterilization	55 (34.3)	372 (53.6)	0.076
Condom	50 (39.4)	237 (34.1)	
Others	22 (17.3)	85 (12.2)	
**Time since assumed infection (yrs)**	7.1 ± 4.1	5.9 ± 3.4	0.001 ^#^
**Receiving HAART**	74 (58.3)	321 (46.3)	0.013
**Baseline CD4 count < 350 cells/μL**	85 (66.9)	364 (52.4)	0.003
**CD4 count nadir (cells/μL)**	188.9 ± 142.7	239.9 ± 182.8	0.000

Data are mean ± standard deviation or n (%)

# Mann-Whitney U test

ASCUS+, atypical squamous cell of undetermined significance or higher grades;

NIL, negative for intraepithelial lesion; HAART, highly active antiretroviral therapy

### Cumulative incidence of ASCUS+

**Figure 1:**
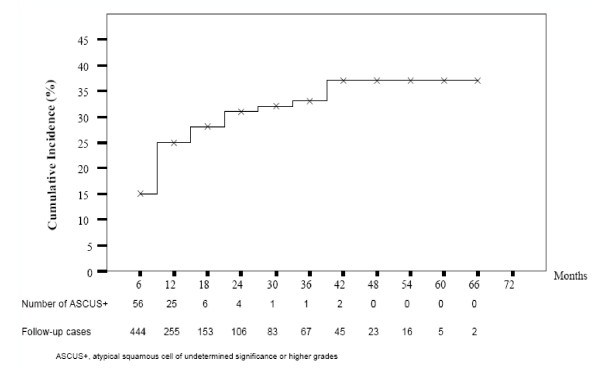
**Cumulative incidence of ASCUS+**.

Of the 444 women who had NIL at initial Pap smear screening and came for the second test, the cumulative incidence of ASCUS+ was 15%. The cumulative incidence was gradually increased in the first 7 visits to 37% and tended to plateau in the last 4 visits (Figure 1). There was a median follow-up time of 12 months when the first ASCUS+ was detected. The mean and median number of Pap smears per patient was 2.7 and 2.0, respectively. None of the patients got pregnant during the follow-up visits in this study. There was no statistical difference between development of ASCUS+ from NIL in terms of age, number of abortions, contraceptive methods, receiving HAART, assumed duration of HIV infection, or CD4 count nadir level. However, the proportion of multipara women and women with CD4 count < 200 cells/μL showed statistically significant differences between the two groups (84.2% vs. 72.8%, *p *0.022; 37.9% vs.20.9%*, p *= 0.016, respectively). In addition the proportion of women with CD4 count < 350 cells/μL was still significantly higher (62.1% vs.48.4%, *p *= 0.018)(Table 3). By multivariate correlation analysis, adjusted for multiparity women and CD4 count < 350 demonstrated that the CD4 count <350 cells/μL was the only significant risk factor for developing subsequent ASCUS+ (*p *= 0.043).

**Table 3:** Comparison of the baseline characteristics of women with subsequent ASCUS+ and NIL who had a NIL at first Pap smear

	ASCUS+ (N = 95)	NIL (N = 349)	*P*
**Age (yrs)**	30.0 ± 8.7	30.1 ± 7.0	0.901
**Gravidarum ≥ 1**	85 (89.5)	299 (85.7)	0.337
**Parity ≥ 1**	80 (84.2)	254 (72.8)	0.022
**Abortion ≥ 1**	23 (24.2)	115 (33.0)	0.103
**Contraception**			
Tubal sterilization	60 (63.2)	198 (56.7)	0.187
Condom only	28 (29.5)	101 (28.9)	
Others	7 (7.4)	50 (14.3)	
**Time since assumed infection (yrs)**	6.1 ± 2.7	5.9 ± 3.0	0.661 ^#^
**Receiving HAART**	52 (54.7)	160 (45.8)	0.124
**Baseline CD4 count < 350cells/μL**	59 (62.1)	169 (48.4)	0.018
**CD4 count nadir (cells/μL)**	206.3 ± 149.4	244.9 ± 189.1	0.067 ^#^

Data are mean ± standard deviation or n (%)

# Mann-Whitney U test

HAART, highly active antiretroviral therapy; ASCUS+, atypical squamous cell of undetermined significance or higher grades; NIL, negative for intraepithelial lesion

### Colposcopic and histologic diagnosis of ASCUS+

Of the 95 women with subsequent ASCUS+, 16 (16.8%) had ASCUS, 1 (1.1%) had ASC-H, 60 (63.2%) had LSIL and 18 (18.9%) had HSIL (data not shown). Seventy six women were able to undergo colposcopic investigation and 19 of them (25.0%) were subsequently colposcopically-diagnosed CIN II-III (Table 4). Nineteen of twenty-four women who were colposcopically diagnosed CINII-III and/or cytologically diagnosed HSIL were able to undergo loop electrical excision procedure of cervix (LEEP) or cold knife conization at this hospital. There were histological assessments which confirmed CIN II-III in 12/19 (63%) and invasive squamous carcinoma in 1/19 (5.3%).

**Table 4:** Colposcopic results in women with abnormal Pap smears

		Colposcopic results n(%)		
		
Abmornal Pap smears	N	Normal	HPV/CIN I	CIN II-III
**ASCUS**	14	4 (28.6)	9 (64.3)	1 (7.1)
**LSIL**	46	3 (6.5)	36 (78.3)	7 (15.2)
**HSIL**	16	0	5 (31.3)	11 (68.7)
**Total**	76	7(9.2)	50 (65.8)	19 (25.0)

ASCUS, atypical squamous cell of undetermined significance

LSIL, low grade squamous intraepithelial lesion

HSIL, high grade squamous intraepithelial lesion

HPV, human papilloma virus

CIN I, cervical intraepithelial neoplasia I

CINII-III, cervical intraepithelial neoplasia II-III

## Discussion

This is the first long-term retrospective cohort study of cervical squamous cell abnormalities in HIV-infected Thai women. It is well recognized that women with HIV infections have a higher prevalence, incidence, persistence, and progression of squamous intraepithelial lesions as compared with those without the infection. The prevalence of cervical squamous cell abnormalities and SILs in the present study was similar to that in our previous study[4]. Compared with several previous studies[5-8], the result of Pap smear screenings from the present study showed a slightly lower prevalence (15.4% vs.20%-40%). In addition to the different backgrounds, this may be due to the fact that the majority of participants in this study were either ante-partum, or immediate postpartum.

Interestingly, the cumulative incidence of ASCUS+ in the present study gradually increased to 37% in the first 3.5 years of follow-up appointments (first 7 times) and tended to plateau in the last 2 years. In addition, one woman with cytological diagnosis of HSIL and with a histologic diagnosis of CINIII was diagnosed from the Pap smear at her seventh follow-up appointment. Comparable with this study, a previous study found that among HIV- infected women whose initial Pap smear was negative for intraepithelial lesion (NIL), about 20%-35% of them would develop cytologic abnormalities over 3.0-5.5 years[3]. This supports the fact that there is a high rate of false negative Pap smear results among HIV-infected patients, as mentioned in a previous study[16]. The findings prompted us to reconsider the appropriate interval of Pap smear screenings for HIV-infected women as recommended by The US Public Health Service and The Infectious Disease Society of America in our setting. Our population might require more than two normal semi-annual Pap smear before shifting to annual cytologic screening. As the present study had high drop-out rate in the first 2 years, we believe that the real incidence of ASCUS+ might be higher. Previous studies have base analysis on less frequent Pap smears than ours; some of them did not pay sufficient attention to the lag period which is supposed to play an important part in cervical carcinogenesis.

Many studies demonstrated that women with a CD4 count < 200 cells/μL were at particular risk of cervical cell abnormalities[4,16-19]. This means that immunological status also plays a crucial part in cervical carcinogenesis. We found that the women with ASCUS+ had significantly higher proportions of receiving HAART than those with NIL. This is the most likely reflection that these women had an underlying poor immune status by their own merit without any correlation of HAART. The CD4 count cut-off point that we used to predict cumulative incidence of ASCUS+ was 350 cells/μL, which is compatible with a study from Brazil[19]. Recently, the Thai Ministry of Public Health initiates HAART for all HIV-infected patients who have CD4 counts at this level or lower. The CD4 counts < 200 cells/μL seem to be far too low to detect new ASCUS+ cases and may be too low for basing a decision to initiate HAART regimen. However, we did not look at the change of CD4 count and its impact on cumulative incidence of ASCUS+.

A well-designed study demonstrated that the incidence of SILs increased with time, especially the ones with lower CD4 count and oncogenic HPV infection[17]. In addition, a study from Italy with 132 HIV-infected women who had invasive cervical cancer (ICC) showed that the interval between the first HIV-positive test and invasive cancer diagnosis was longer than 10 years in almost half of the women[20]. We also found that the assumed duration of HIV infection and the CD4 count nadir level were associated with a high prevalence of ASCUS+ from the initial Pap smear. However, they were not associated with the cumulative incidence of ASCUS+.

In the present study only 0.1% (1/821) of HIV-infected women had invasive squamous cell carcinoma. This woman had a baseline CD4 count of 148 cells/μL and had a 14-year duration assumed HIV infection. She had a cytologic HSIL at the third Pap smear and colposcopic diagnosis of HSIL. With a lower incidence of invasive squamous cell carcinoma, our study may not represent the whole picture of HIV-infected women in Thailand - since the incidence in this study was lower than the figure reported from a previous study conducted in Thailand[21]. A high number of women were already on HAART at commencement of the study. HAART might have beneficially protective on preventing cervical carcinogenesis[22,23]. More important reasons were the early detection by a regular semi-annual check-up, the development of health education, and the growth of trust between health care providers and patients. Smoking was not included in the baseline characteristics because almost all Thai women were not current or ex-smokers.

Several studies are currently investigating the benefits of adding HPV DNA tests to improve screening for cervical lesions and cancer - as screening for oncogenic HPV types is a more sensitive predictor of high grade squamous intra-epithelial lesions. Overall, the HPV test had a higher sensitivity among HIV-infected women as compared with HIV-uninfected women. One study in Thailand reported that the prevalence of high risk HPV infection in Thai HIV-seropositive women was 38.6%[21], which was lower than that found in American (83.2%) and Brazilian women (44.5%)[24,25]. However, HPV DNA testing is not a routine screening test in this clinic due to its high cost. In addition, the specificity for cervical lesions of the test was low in HIV-infected women, resulting largely from a very high prevalence of HPV infection in women without cervical lesions. Thus, a HPV test may not provide benefits for cervical surveillance in the setting of HIV, because of its low specificity and poor predictive value[26,27].

In 1999, Holcomp, et al. demonstrated the significance of ASCUS in HIV-infected women by comparing cytological and histological results[28]. They found that 32% of ASCUS had histological cervical intraepithelial neoplasia (CIN). As a result, they suggested that early colposcopy should be considered in HIV infected women with ASCUS. There were a total of 16 women with ASCUS in our study, 14 were able to undergo colposcopy and 10 had colposcopic diagnosis of SILs (9 women with HPV/CIN I and 1 woman with CIN II-III) (Table 4). Although our study demonstrated that there was a high incidence of colposcopic diagnosis of SILs in women with ASCUS, only one woman in this group had colposcopic diagnosis of CIN II-III which had to be confirmed by tissue diagnosis. Since most of them had colposcopic diagnosis of HPV/CIN I and had a low socio-economic background, it was unlikely that biopsies with pathological reports in these cases could be met.

There were a number of limitations in the present study that warranted mentioning. (1) The primary outcome was mainly the surrogate outcome of cervical cancer. Even though the incidence of abnormal lesions was high, these were mainly low grade lesions which could be regression, especially in younger women[29]. (2) False negative Pap smear was not included in the scope of the present study and this might have impact the findings as a previous study found a high false negative Pap smear rate in HIV-infected women with CD4 count < 500 cells/μL[16]. (3) The lack of viral load, colposcopy with tissue biopsy could not be performed in all cases; instead, a 'see and treat' technique was applied in order to decrease costs. (4) Due to the government universal coverage program and limited seats at Siriraj Hospital, many participants, especially a number in our study who were either ante-partum, or immediate post partum, were required to follow-up at their registered hospitals causing 250 women to be lost to follow up by the 6 month visit (36%) and a further 133 (20%) lost at 12 months. This is a loss of over 50% of study participants in the first year of follow-up. In addition, an effort to try to establish some relationship between health care providers and HIV- infected women was very difficult because of stigmatization of HIV. As a consequence, the rate of follow-up was quite low leading to the potential biases, such as survivorship bias/retention in care. In addition, other AIDS-indicated conditions and established risk factors of cervical cancer were not accounted for and there was one patient who died from an opportunistic infection during the follow-up period.

## Conclusions

There are high prevalence and cumulative incidence of ASCUS+ in HIV-infected Thai women. With a high lost-to-follow-up rate, an appropriate interval of Pap smear screening cannot be concluded from the present study. Nevertheless, the HIV-infected Thai women may require more than two normal semi-annual Pap smears before shifting to routinely annual cytologic screening.

## Competing interests

The authors declare that they have no competing interests.

## Authors' contributions

AC and CC took initiative in developing the research project, and drafted the manuscript. AC participated in the design of the study. AC and CK participated in the data analysis. AC, CC and MT participated in the writing of the manuscript. All authors read and approved the final manuscript.

## Pre-publication history

The pre-publication history for this paper can be accessed here:

http://www.biomedcentral.com/1471-2334/11/8/prepub
